# Optimization of Carotenoids Production from *Rhodotorula* sp. Strain ATL72 for Enhancing Its Biotechnological Applications

**DOI:** 10.3390/jof8020160

**Published:** 2022-02-06

**Authors:** Amira Dyaa, Hoda Soliman, Ahmed Abdelrazak, Bassem N. Samra, Ebtihal Khojah, Atef F. Ahmed, Mohamed A. El-Esawi, Ashraf Elsayed

**Affiliations:** 1 Botany Departement, Faculty of Science, Mansoura University, Elgomhouria St., Mansoura 35516, Egypt; amira_dyaa@yahoo.com (A.D.); hoda@mans.edu.eg (H.S.); Ahmed_bt@mans.edu.eg (A.A.); 2 Department of Biology, College of Science, Taif University, P.O. Box 11099, Taif 21944, Saudi Arabia; b.elsayed@tu.edu.sa (B.N.S.); atefali@tu.edu.sa (A.F.A.); 3 Department of Food Science and Nutrition, College of Science, Taif University, P.O. Box 11099, Taif 21944, Saudi Arabia; eykhojah@tu.edu.sa; 4 Botany Department, Faculty of Science, Tanta University, Tanta 31527, Egypt

**Keywords:** carotenogenic yeast, Plackett–Burman, central composite design

## Abstract

*Rhodotorula* yeasts which are known as carotenogenic yeasts have a great industrial value due to their ability to produce carotenoids. In particular, the isolated yeast *Rhodotorula* sp. (strain ATL72) has been reported to be a promising producer of high concentrations of carotenoids. A combination of central composite design (CCD) and Plackett–Burman (PB) design was used to optimize carotenoids produced by this yeast. The optimum production of carotenoids was completed when the yeast was grown in a production medium composed of 3.7 g/L malt extract, 7.7 g/L fructose, 9 g/L urea, 35 g/L NaCl, and 1 g/L yeast extract at 27.5 °C, pH 6.7, and 180 rpm. Two batch runs in 1 L and 7 L bioreactors were conducted which increased the productivity of carotenoid concentration from 21.5 mg/L after 98 h of incubation at the level of the shake flask to 229.9 mg/L after 47 h of incubation at the level of 7 L bioreactor. The carotenoid pigment was extracted in dimethylsulfoxide (DMSO), acetone, petroleum ether, and sodium chloride, and subsequently identified and characterized using UV-visible scanning, thin layer chromatography, and gas chromatography/mass spectrometry.

## 1. Introduction

Microorganisms, such as bacteria and yeast, are considered as a vital source of biopigments like carotenoids [1,2]. Animals and higher plants are also able to synthesize carotenoids [3]. Carotenoids are a group of natural pigments present in egg, fish, fruits, and vegetables and can be synthesized by different organisms such as *Rhodotorula* [4,5]. The yellow, orange, pink, or red carotenoids are secondary metabolites produced by different organisms including yeast *Rhodotorula* sp. [6].

There are a variety of natural sources of carotenoids. Photosynthetic organisms such as cyanobacteria and algae and non-photosynthetic organisms such as yeast, bacteria, and fungi are good natural sources of carotenoids. Additionally, aquatic animals, microalgae, and all of the colorful vegetables and fruits are a major natural sources of carotenoids [7]. On the other hand, carotenoids are synthesized chemically, through different chemical reactions [8].

Carotenoids have a significant medical, industrial, food and pharmaceutical importance due to their antioxidative function, which increases their activity against chronic and cancer diseases and also enhance immunity and vision [9].Carotenoids were reported to have antioxidant, anticancer, and antiobesity effects [10,11]. Synthetic carotenoids have a deleterious effect on the environment and public health compared to natural pigments. On the other hand, carotenoids from higher plants and animals suffer from uncontrolled issues related to the weather and geographical problems [12], while the microbial carotenoids are considered the optimal eco-friendly and economic alternative. 

Globally, the demand for using carotenoids as naturally derived pigments is increasing. To fulfill these demands, new biotechnological applications should be used. Scaling up the production of any microbial products can be performed precisely by detecting the optimum natural conditions. Recent studies aim to use high-carotenoid production yeasts such as *Rhodotorula*, agro-industrial wastes, and bioreactor design to cover the worldwide demand of naturally synthesized carotenoids [13].

Over the years, different methods of optimization were developed and each one pretended to be the perfect method. Those methods include Build-Test-Fix [14], One Factor at a Time (OFAT) [15], and Design of Experiments (DOE) [16]. There are two types of DOE experiments. First, factorial designs that test the effects of all factors and the interaction between them by factorial experiment which includes a two-level full factorial design, two-level fractional factorial design [17], and screening factorial design [18]. Second, Response Surface Methodology (RSM) that determines the optimal settings of the experimental factors and includes central composites, Box–Behnken designs, 3-level factorials, and Draper–Lin design. RSM explains the interaction effect between the factors in a fermentation process [19].

Response Surface Methodology (RSM), Plackett–Burman design (PBD), and the bioreactor optimized the production of carotenoids from *Rhodotorula* ATL72 [20,21]. This study aimed to use the biotechnological process as optimization and a bioreactor to enhance the production of carotenoids by *Rhodotorula* ATL72.

## 2. Materials and Methods

### 2.1. The Strain and the Produced Pigment

The used strain has been previously isolated and identified by the authors and it was found to be more closely related to *Rhodotorula bloemfonteinensis* (EU075187) based on 18S rRNA gene analysis. This locally isolated strain has been found to be a potential high producer of the pink carotenoids pigment [22].

### 2.2. Optimization of Various Physico-Chemical Parameters Using Central Composite Design

CCD design was utilized to optimize variable factors such as pH, temperature, and salinity to determine the optimum conditions for the cultivation at the level of the shake flask, through defining the main effects and the interaction between the previous factors on the yeast growth and the production of carotenoids [23]. Each variable in the CCD matrix has five levels, which were chosen according to previous studies [22,23] that showed the effects of these variables on the production of carotenoids, as shown in Table 1.

To test the effect of each variable, enriched broth media was used which consisted of yeast extract 5 g/L, glucose 10 g/L, and sea water. The experiments were performed in a 50 mL flask and each one was incubated at 180 rpm in a shaking incubator according to what is displayed in the matrix in Table 1. The growth rate was detected by recording O.D_600_, while the carotenoid production was detected by calculating the carotenoid concentration.

### 2.3. Screening the Medium Components by Placket–Burman Design

The main aim of using Placket–Burman design is to screen the important components of the media concerning their individual major effects not the effects that result from the interaction between different medium components [24].

Thirteen different kinds of relatively cheap available components of the media were utilized, including glucose, peptone, sucrose, urea, glycerol, malt extract, fructose, molasses, ammonium chloride, whey, yeast extract, potato extract, NH_4_NO_3_, and one dummy factor to measure the standard error of the design. All chemicals were purchased from Sigma-Aldrich Egypt, each factor was utilized at a maximum concentration (+) and minimum concentration (−) where the maximum level of all the tested variables is 1 g/L and the minimum value to be tested is the complete absence of the variable. 

The major effect of each factor was determined by the (Equation (1)).

E(Xi) = 2[ΣYi+ − Yi−]/N(1)

where E(Xi) is the effect of the tested factor, Yi+ and Yi− are the calculated responses.

The significance level (*p*-value) for each factor was detected using Student’s *t*-test

t(Xi) = E(xi)/SE(2)

where (SE) is the standard error for the factors. Any factor with *p*-value < 0.1 is significant at the 90% confidence level.

The relationship between the significant factors and the response within 2-level factorial design depends on first order polynomial order (Equation (3)) as no interactions could be calculated by this screening design.

Y = β0 + ΣβiXi (1 = 1, 2, … … …, K) (3)

where Y is the calculated response, β0 is model intercept, βi is the regression coefficient for each corresponding variable, Xi is the corresponding variable, and K is the number of variables [25].

The experiments were performed in Erlenmeyer flasks containing 50 mL of basal media (yeast extract 1 g/L and NaCl 35 g/L) besides the components of each trial and incubated in optimized conditions of 180 rpm and 27.5 °C for 96 h. Responses were estimated in terms of OD_600_ and carotenoid content (mg/L). 

### 2.4. Optimization of the Production Medium by Central Composite Design

After utilizing PBD, to detect the most potential components of the production medium, the optimum concentration of each factor was defined using CCD. The matrix of the central composite design has 5 levels for each factor, 6-star points and center points to detect the curvature. The CCD studied the major effect of each factor and the interaction between them. 

A second-order polynomial model was used for the prediction of the optimum components of the production medium for carotenoid production, as shown in (Equation (4)):

Y = β0 + ΣβiXi + Σβiixii + ΣβijXij(4)

where βi represents the regression coefficient of each variable, βii represents the regression coefficient for square effects and βij represents the regression coefficient of the interactions. Using Design Expert 8.0 statistical package (StatEase, Inc., Minneapolis, MN, USA), an analysis of variance (ANOA) was completed.

CCD was used to optimize the potential components of the media through 25 trials with 3 variables and 5 levels as shown in Table 2 [26].

Factors with a major positive effect on both growth of yeast and carotenoid production were malt extract, urea, and fructose. Each factor was studied at a maximum concentration (+) and at a minimum concentration (−) where the maximum concentration is expressed as 9.3 g/L and the minimum concentration is expressed as 2.6 g/L.

### 2.5. Time Course of Carotenoid Production by Rhodotorula ATL72

The time course for the growth and carotenoid production was studied by culturing the yeast on the optimized media. In total, 200 mL of overnight seed culture was inoculated with O.D_600_ 1 nm and incubated at 27.5 °C, 6.7 pH, and 180 rpm for 72 h. Twenty flasks with 100 mL working volume for each were inoculated with 10 mL of the seed culture and incubated at 27.5 °C and 180 rpm for 98 h. Samples were withdrawn at different time intervals for analysis [27].

### 2.6. Batch Run in 1 L Bioreactor

**Figure 1:**
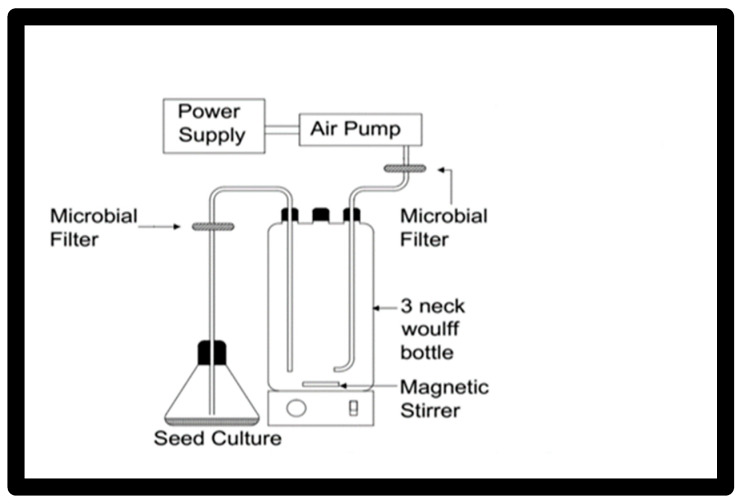
The 1 L bioreactor used in the batch run for carotenoid production.

A culture system in Figure 1 using a 1 L flask, 3-neck Woulff bottle was used for scaling up the growth and the carotenoid production for the yeast *Rhodotorula* ATL72. Two lines were fitted at the shoulders of the flask, one for aeration and the other for inoculation and harvesting. Each line was supplied with a microbial filter to prevent contamination. Air flow was supplied using an air pump and sparged to the system by passing through a microbial filter prior to entering into the media. The temperature was adjusted by placing the flask in the incubator at 27.5 °C. The agitation speed was adjusted using a magnetic stirrer at a speed of 250 rpm. The seed culture of 100 mL was prepared from freshly cultured broth, which had the exact concentrations of the nutrients of the production medium. The concentrations of the nutrients in the production medium were the following (g/L): malt extract 3.7, fructose 7.7, urea 9, yeast extract 1, and NaCl 35 and inoculated in 1000 mL of the optimized media. Samples were withdrawn at different time intervals for calculation of the growth rate (optical density at 600 nm) and carotenoid content (mg/L). Figure 1 displays the proposed bioreactor design.

### 2.7. Batch Run in the 7 L Bioreactor

A batch run was performed using an Eppendorf–New Brunswick 7 L Rushton turbine Stirred Tank Bioreactor with a working volume of 3 L. The bioreactor was set after sterilization. The seed culture was inoculated by a loopful of fresh plate of the yeast, of age 48 h, and maintained at 27.5 °C, pH 6.7, and 180 rpm. The batch run occurred at 27.5 °C, 180 rpm, uncontrolled pH, and controlled dissolved oxygen (D.O) at 10%. For calculations of the growth and carotenoid content, samples were withdrawn at constant time intervals.

A total of 700 mL of the seed culture was prepared, which had the exact concentrations of the nutrients of the production medium. The concentrations of the nutrients in the production medium were the following (g/L): malt extract 3.7, fructose 7.7, urea 9, yeast extract 1, and NaCl 35.

### 2.8. Extraction and Determination of Carotenoids

Extraction of carotenoids from yeast was performed according to Sedmak et al. [28]. The extraction was completed as follows: the yeast biomass was harvested by centrifugation and washed with distilled water three times. Then, the extraction was performed as follows: 5 mL of dimethylsulfoxide (DMSO), acetone, petroleum ether, and 20% NaCl were added serially to the harvested pellet with vortexing [29,30,31].

The total carotenoid was determined by measuring the optical density according to [28,30]. The total carotenoid content from the yeast pellet was expressed as volumetric carotenoids (mg/L). The extension coefficient ($E_{1\%}^{1cm}$) 2680 was used according to the following equation:

$$\mathrm{Volumetric}\;\mathrm{carotenoids}\;(\mathrm{mg}/L)=\frac{A\;\cdot \;V\;\cdot \;10^{6}\;}{E_{1\%}^{1cm}\;\cdot \;100}$$

where:

*A*: absorbance at 490, *V*: total volume: extinction coefficient

All the above procedures were performed under low light to avoid pigment degradation [26,32,33].

## 3. Results

### 3.1. Optimization of Various Physico-Chemical Parameters Using CCD

The carotenoid production and the growth rate were affected by vital factors such as temperature, pH, and salinity and their levels were determined according to previous research. Therefore, the optical density of the growth and carotenoid concentration (expressed as OD_600_ for growth and mg/L for carotenoid concentration) were used as calculated responses to be optimized. Twenty different experiments were performed, each one with its characteristic conditions, in a shaking flask at 180 rpm and the responses were measured and reported for each experiment. Table 3 shows the matrix and responses for the CCD design.

The maximum amount of the carotenoid’s concentration was obtained on run numbers (16 and 18) achieving 25.14 mg/L. Meanwhile, the minimum amount was obtained on run number (9) where the concentration of carotenoids was reduced to 1.79 mg/L. The maximum growth of the isolated yeast *Rhodotorula* ATL72 was obtained on run numbers (16 and 18) achieving 0.866 at O.D_600_. Meanwhile, the minimum growth was obtained on run number (9) where the growth was reduced to 0.045 at O.D_600_. Interactions between factors and linear variance, in addition to analysis of the quadratic effect, are shown in Table 4. The factors were considered to be significant when *p*-value < 0.1 and marked in Bold. The responses of the experiment were subjected to the analysis of variance and parameter estimates and results are summarized in Table 4.

ANOVA analysis showed that the most influential factor on the growth rate of the yeast is temperature with a *p*-value of 0.009 while the main effects of both salinity and pH were found to be insignificant with *p*-values of 0.242 and 0.916, respectively. 

**Figure 2:**
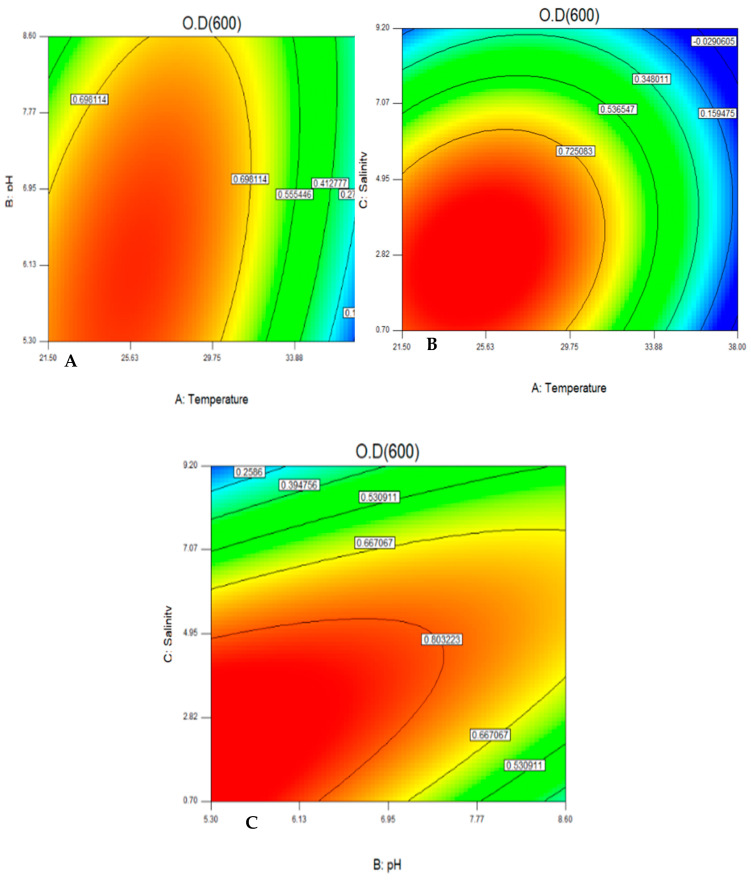
Color contour plot for the interaction between pH and temperature (**A**), salinity and temperature (**B**), and salinity and pH (**C**) for optimal growth of the yeast.

**Figure 3:**
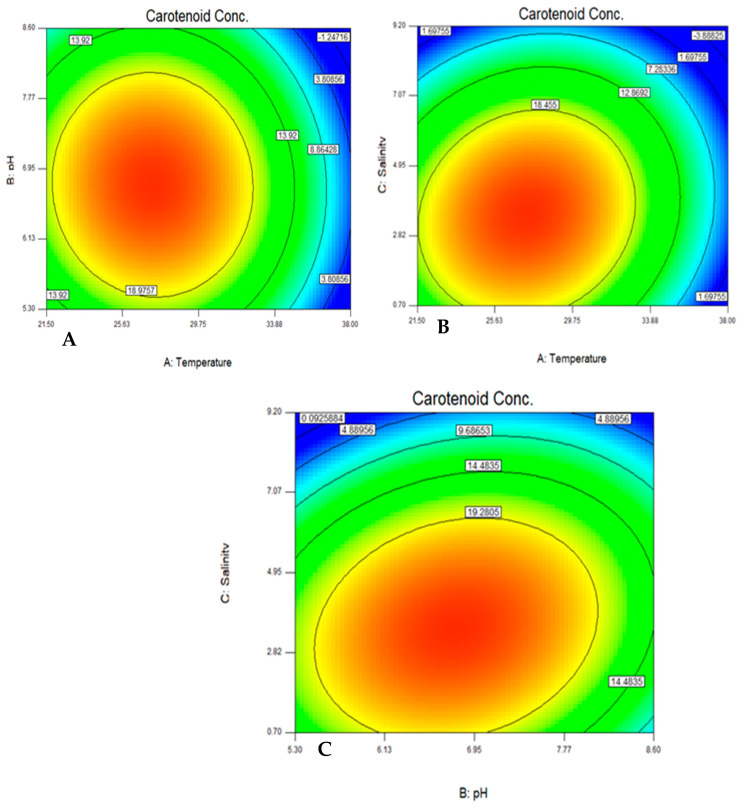
Color contour plot for the interaction between pH and temperature (**A**), salinity and temperature (**B**), and salinity and pH (**C**) for optimal production of carotenoids.

The quadratic effects of both temperature and salinity were found to be significant factors affecting the yeast growth while the main effects of both temperature and salinity and the quadratic effect of temperature, salinity, and pH were found to be significantly affecting the carotenoid production. The interactions between each of the tested variables are shown in the form of contour plots (Figure 2 and Figure 3). 

Optimum conditions for carotenoid production were temperature 27.5 °C, pH 6.7, and salinity 3.5% leading to 25.14 mg/L. 

### 3.2. Screening of the Medium Components by Placket–Burman Design

PBD trials were utilized to define the most significant components for production of carotenoids and yeast growth, which was subsequently optimized by Response Surface Methodology to detect the optimum combination and construct a mathematical model that could be used in the prediction process. Thirteen different components of the media were screened and each one was investigated at high level (+) and low level (−) in addition to a dummy factor which was used to estimate the standard error of the experiment. Twenty experiments, each one with its characteristic combination of the tested factors, were performed and values of growth and carotenoid conc. were calculated as in Table 5.

The maximum production of the carotenoids was 5.1 (run 6) and 4.9 mg/L (run 9), while the maximum growth of yeast was 2.58 (run 4) and 2.37 (run 9). The minimum production of carotenoids was 1.7 mg/L (run 7) and for the growth was 0.095 (run 7). Table 6 shows the analysis of the responses, estimates of the parameter, and results. The analysis of the responses declared that urea, malt extract, and fructose have a statistically positive effect on growth of the yeast while malt extract and potato ex. have a statistically positive effect on the production of carotenoids as explained in Table 5. Statistical analysis of the responses, using Minitab 16, defines the factors with positive major effects on both the growth of the yeast and carotenoid conc. at confidence level of 90%. A significant negative effect means that the factor has a major effect in low concentrations. Meanwhile, a significant positive effect means that the factor has a major effect in high concentrations.

The interaction between factors and calculated responses can be mathematically expressed using first order polynomial models as follows:

Y1 = 0.007435 × Urea + 0.014175 × Malt extract + 0.009315 × Fructose + 0.002665 × Yeast extract − 0.000525 × NH_4_Cl

For Carotenoid conc. (mg/L)

Y2 =Malt extract − 0.285 × Yeast extract − 0.0044 × Whey + 0.445 × Potato Ex.

**Figure 4:**
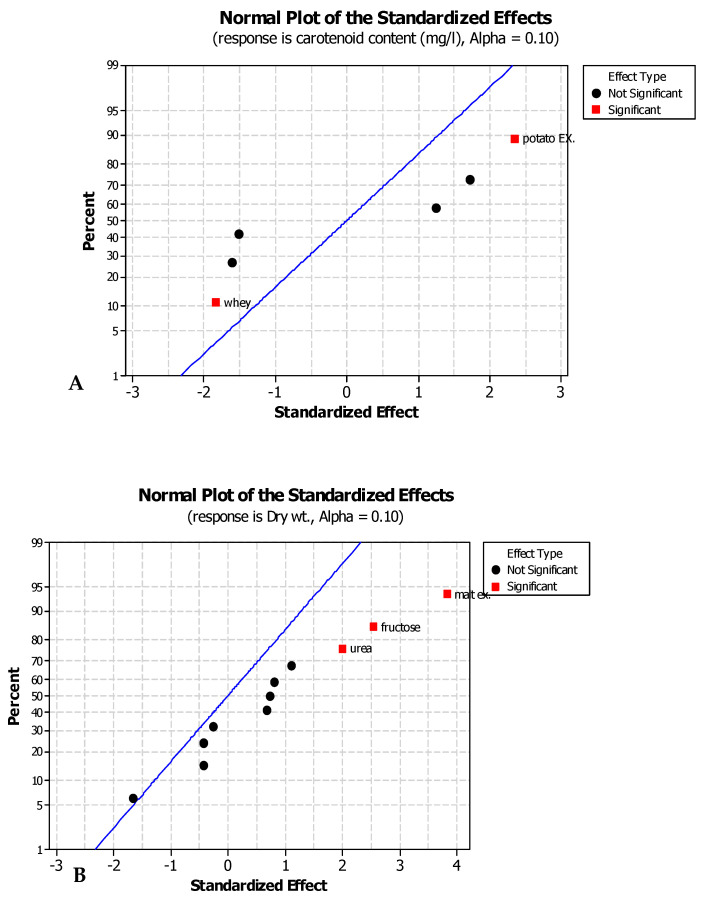
Normal plot showing the significant effect of each variable on (**A**) carotenoid conc. and (**B**) dry weight.

The standardized effect was drawn in a normal plot, as shown in Figure 4, demonstrating that the factors that are statistically significant have a positive effect and are found on the right of the line, whereas those found on the left have a negative effect. 

**Figure 5:**
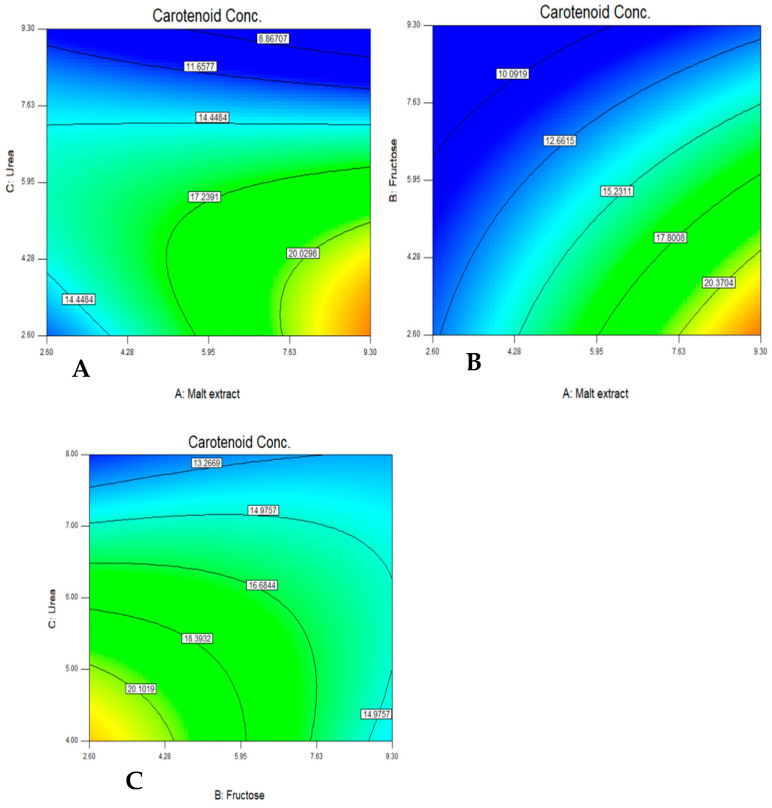
Color contour plot showing the optimum interaction between urea and malt extract (**A**), fructose and malt extract (**B**), and urea and fructose (**C**) for maximum carotenoid production.

Malt extract, fructose, and urea have a statistically significant positive effect on the growth of the yeast with *p*-values < 0.1. Potato extract has a positive effect on the carotenoid conc. with *p*-values < 0.1 while whey has a negative significant effect as represented in Figure 5. 

### 3.3. Optimization of the Production Medium by Central Composite Design

Malt extract, fructose, and urea were found to have a positive significant effect on growth of the yeast as reported during the screening PBD. To define the optimal medium components, CCD was performed as shown in Table 7.

The main goal of the optimization process is generating a cheap and available medium which decreases the production cost of carotenoids and increases its productivity.

The experimental responses were subjected to analysis of variance and parameter estimates and results are summarized in Table 8.

Upon applying the ANOVA, the following terms were found to have a statically significant effect on the growth of the isolate including urea, the quadratic effect of malt extract, and the quadratic effect of urea. The color contour plot shows the optimum concentrations of malt extract, fructose, and urea on the optimal production of carotenoids as shown in Figure 5.

For urea, the optimum concentration ranges between 2.5 and 4.2 g/L and for malt extract, the range is between 8 and 9.3 g/L. For fructose, the optimum concentration ranges between 2.6 and 3.5 g/L. For malt extract, the range is between 8.3 and 9.3 g/L. For urea, the optimum concentration ranges between 4 and 4.5 g/L and for fructose the range is between 2.6 and 3.2 g/L, as shown in Figure 5. 

#### Model Validation

The maximum optical density and carotenoid conc. could be achieved using another combination of other concentrations of malt extract, fructose, and urea, according to the model prediction.

The predicted experiments for the optimal combination between the variables are shown in Table 9.

The predicted experiments were verified experimentally as shown in Table 9, the maximum growth and carotenoid conc. occurs at 3.7, 7.7, 9, 1, and 35 g/L of malt extract, fructose, and urea, respectively, leading to maximum carotenoid production of 24.6 mg/L and 3.77 O.D_600_ for growth.

### 3.4. Time Course of Carotenoid Production by Rhodotorula ATL72

A total of 100 mL of the developed optimized production medium was inoculated by the isolate *Rhodotorula* ATL72 and incubated at 27.5 °C, at 6.7 pH, and 180 rpm. Samples were withdrawn at time intervals every two hours in order to monitor the growth and productivity of the isolate under investigation when growing on the proposed developed medium.

**Figure 6:**
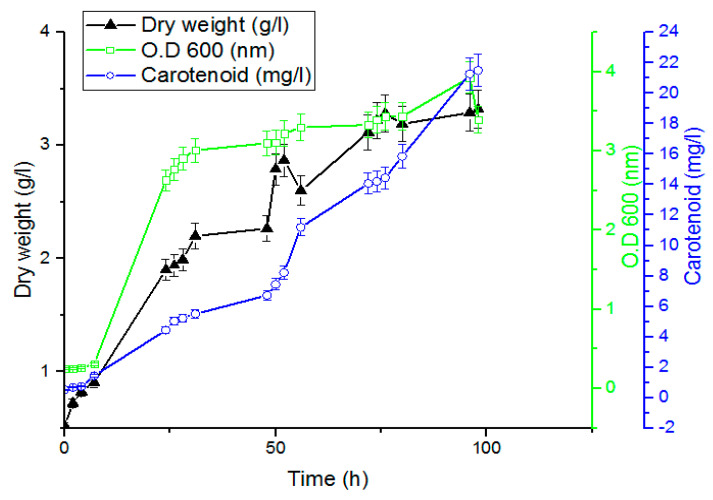
Yeast growth and carotenoid production in relation to incubation time (h) at shake flask level showing the maximum production of carotenoids (21.5 mg/L) after 98h (bars represent standard deviation of the mean).

The analysis of the samples revealed that carotenoid production is not a growth-associated product as the carotenoid production increases the growth stopped at the stationary phase and the maximum amount of carotenoid obtained after 98 h was 21.5 mg/L, as shown in Figure 6.

### 3.5. Bioreactor Batch Runs

As a crucial step toward the scaling up and development of a suitable industrial process for carotenoid production by the isolate *Rhodotorula* ATL72, the growth and productivity behavior of the isolate within the bioreactor environment was tested. Two subsequent reactors were tested at two different scales (1 L and 7 L reactors).

#### 3.5.1. Batch Run in the 1 L Bioreactor

A 1 L flask, 3-neck Woulff bottle of 1 L working volume was used as the culture system. A seed culture of 100 mL was used for inoculating 900 mL of the optimized media. The conditions were adjusted at 27 °C and 6.7 pH, with an air flow of 2 L/min. 

**Figure 7:**
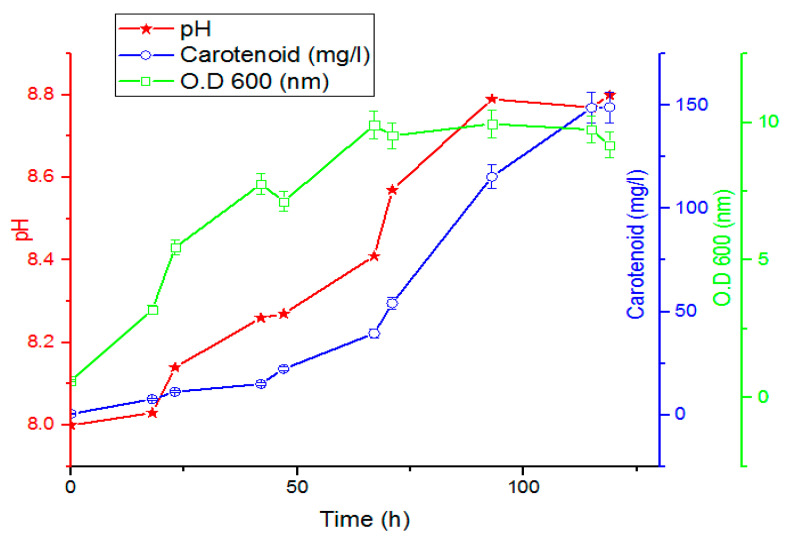
Yeast growth, pH, and carotenoid production in relation to incubation period (h) at 1 L bioreactor level showing a maximum carotenoid conc. value of 149.18 mg/L after 119 h (bars represent standard deviation of the mean).

The results are summarized in Figure 7. The maximum amount of growth obtained was 9.19 O.D_600_, while the maximum achieved production of carotenoids was 149.18 mg/L after 119 h.

The amount of carotenoid was significantly increased when growing the cells in a bioreactor compared to the growth in a shake flask from 21.5 mg/L after 98 h to 18 mg/L after 119 h (approximately 5 times greater carotenoid production was achieved in bioreactor). The significant increase in the amount of carotenoids could be due to the sparging of air and replacing shaking with stirring when cells were grown in a bioreactor.

#### 3.5.2. Batch Run in the 7 L Bioreactor

**Figure 8:**
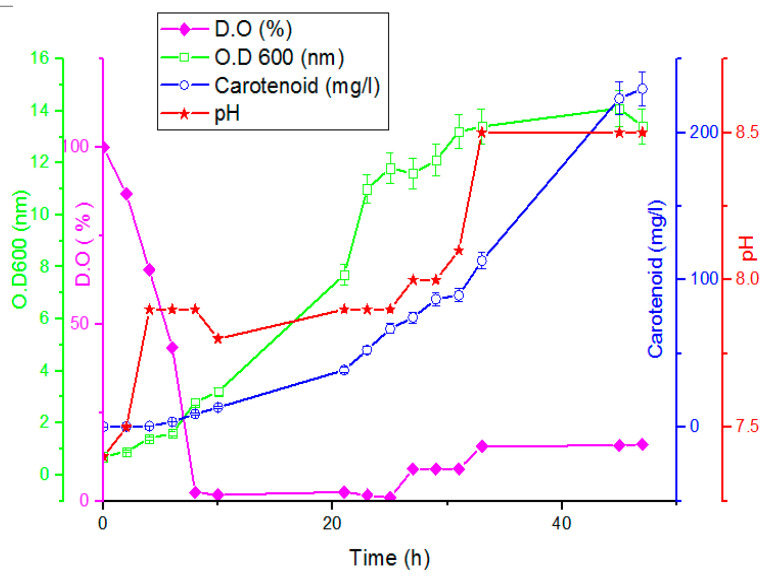
Time trajectory for growth and productivity of *Rhodotorula* ATL72 in a bioreactor on the optimized production medium under precise conditions (27.5 °C, controlled dissolved oxygen 10%, and 180 rpm, bars represent standard deviation of the mean).

A loopful of freshly activated yeast plate of age 48 h was used for inoculating the seed culture and kept at 27.5 °C, 6.7 pH, and 180 rpm in an orbital shaker incubator for 24 h. Once the seed culture achieved 0.7 O.D_600_, it was used to inoculate the 7 L bioreactor. The conditions of the bioreactor batch run were temperature 27.5 °C, agitation 180 rpm, pH uncontrolled, and dissolved oxygen controlled at 10% (air was sparged at a speed of 2 L/m). The samples were taken at constant time intervals for the calculations of the growth (optical density at 600 nm) and the carotenoid conc. (mg/L), and the results are illustrated in Figure 8, as the maximum production of carotenoid was 229.9 mg/L that was achieved after 47 h inoculation in the bioreactor.

The production medium used for the batch run contained (g/L) malt extract 3.7, fructose 7.7, urea 9, NaCl 35, and yeast extract 1.

Under these experimentation conditions, the D.O decreased by increasing the yeast growth (OD_600_) and carotenoids started to appear after 8 h of inoculation and reached the maximum growth value of 13.4 O.D_600_ and maximum carotenoid conc. value of 229.9 mg/L, after 47 h inoculation in the bioreactor.

## 4. Discussion

In recent years, the interest in natural pigments has increased worldwide as a safe alternative to synthetic ones. Hence, seeking a natural, economical, and safe alternative for synthetic carotenoids is a must. The production of carotenoids varies between species in the genus *Rhodotorula* and is affected by the environmental conditions and the medium components [31]. This work highlights the factors affecting the production of carotenoids and scaling up of both carotenoid production and cellular growth.

*Rhodotorula* ATL72 produces a large amount of the carotenoid pigments in the growth medium. Carotenoids are lipid-soluble pigments and vital membrane components, and they are present within the membrane lipid core [32].

The production of carotenoids in the natural environment is insufficient for commercialization. Thus, microbial production of carotenoids at industrial scale has some criteria for scaling up of the production process, such as consuming several inexpensive carbon and nitrogen sources, pH tolerance, requirement of temperature, developing the cultivation strategies, and utilizing inexpensive downstream process [33].

Design of Experiments (DOE) is an alternate experiment which confirms testing of all variables and their interactions, and the analysis of DOE provides more reliable and complete information than that from One Factor at a Time (OFAT) experiments which causes a decline in the interactions and provides misleading results [34]. 

CCD was applied to study the effect of temperature, pH, and salinity on carotenoid production and yeast growth. Then, Plackett–Burman design (PBD) was used for screening the effective carbon and nitrogen sources for the production of carotenoids. Twenty different experiments, each with their characteristic combination, were applied and the responses to be optimized were evaluated at the end of each trial. The maximum amount of carotenoids was found to be 25.14 mg/L at trial numbers 16 and 18. The results were analyzed by a multi-way ANOVA analysis (Table 4). For indicating the statistical significance of a factor, a *p*-value of <0.1 was used as a cut-off point at 90% confidence level. A *p*-value is significant when equal to or less than α [23]. The main effect of temperature was found to be the most significant factor affecting the yeast growth, while the main effects of both pH and salinity were found to be insignificant factors.

The quadratic effects of both temperature and salinity were found to be significant factors, which are affecting the yeast growth. In the case of factors affecting carotenoid production, the main effects of temperature and salinity and the quadratic effects of temperature, salinity, and pH were found to be the most significant factors. On the other hand, the main effect of pH and the interactions between pH, salinity, and temperature were found to be insignificant factors, as shown in Table 4. 

The color contour plots were generated to show the interaction of the tested variables with the carotenoid production and the optimum combination. The optimum range was found to be 27.5 °C, pH 6.7, and salinity 3.5% NaCl, which agreed with what has been reported by [35]. *Rhodotorula* spp. can grow in a wide range of temperatures (5–35 °C), but the optimum temperature and its activity depend on the species specificity of the yeast [36]. 

*Rhodotorula* ATL72 produced the maximum amount of carotenoid pigments at 27.5 °C and the rate was reduced as the temperature increased, which is in agreement with the mainstream directions in the literature. Most researchers have reported the optimum range of temperature for carotenoid production to be around 20 °C and decreases as the temperature increases to 30 °C. Bhosale and Gadre [37] have reported that β-carotene accumulation was found to be higher at 20 °C and decreases as the temperature increases. On the other hand, Muthezhilan et al. [38] have reported that the optimum conditions of carotenoid production by *Rhodotorula* sp. AMBY109 are temperature 34.29 °C, pH 8.54, and salinity 20.15 ppt. 

The increase in the pigmentation of *Rhodotorula* ATL72 can be attributed to the activity of β-carotene synthase which is more active at low temperature, compared to dehydrogenation and decarboxylation which are responsible for the torulene synthesis [39]. 

The optimum pH for the carotenoid production was found to be 6.7, which is confirmed by Wang et al. [40] who have reported that the optimal pH for the carotenoid production by *Rhodotorula glutinis* (*R. glutinis*) was 6.7 which indicates the preference of the yeast for a slightly acidic pH near 6 [41]. 

There was a significant increase in both the growth and the carotenoid production by *Rhodotorula* ATL72 at 3.5% NaCl which agreed with the findings of Kanzy, Nasr, El-Shazly and Barakat [30] which can be explained by the carotenoids acting as protective agents protecting the cells from free radicals, hence, the biosynthesis of the carotenoids represents a stress response mechanism. Exposure of the cells to mild stress as salt stress leads to overproduction of different substrates and tolerance to higher doses of stress or tolerance to stress by other agents which is known as cross-protection [42], Therefore, the addition of salts induces carotenogensis by the isolate ATL72.

Statistical experimental designs including Plackett–Burman design (PBD) and central composite design (CCD) were used for optimization and enhancement of the growth and carotenoid production. The media that resulted from using these strategies, supported good growth of the yeast and improved the carotenoid biosynthesis at the same time.

Plackett–Burman design (PBD) was utilized for screening 13 relatively cheap and available carbon and nitrogen sources for their effect on carotenogensis and the yeast growth. Three of them (malt extract, fructose, and urea) showed the most positively significant effects on both carotenoid production and yeast growth.

The most significant factor for carotenogenesis and yeast growth was malt extract as the carbon source having high concentrations of carbohydrates. There is a significant effect of malt extract as reported by [43,44]. The second significant factor for carotenoid production and yeast growth was fructose as an additional carbon source as reported by Latha et al. [45,46,47]. The third significant factor was urea as a nitrogen source for carotenoid production and yeast growth as reported by Park et al. [48].

After defining these carbon and nitrogen sources as the most significant medium components for optimizing the carotenoid production, a central composite design matrix was applied to detect the optimum medium compositions and interactions between the variables for optimizing the carotenoid production. The analysis was completed using an ANOVA (Table 6) and it was found that the optimum medium for carotenoid production was as follows (g/L); malt extract 3.7, fructose 7.7, and urea 9. The maximum experimental response for carotenoid production was 24.6 mg/L. The growth conditions and the medium components were found to be vital to carotenoid production by different strains of *Rhodotorula* [44,49,50,51]. No definite medium was reported to be the optimized one, but it varies depending on the growth conditions and the strain. 

The scaling up process from the shake flask to the bioreactor level was completed at two stages; the first stage using a 1 L bioreactor under the optimized medium which increased the productivity to 149.18 mg/L after 119 h. The second stage was completed using a 7 L bioreactor under the optimized medium, showing a remarkable increase in the carotenoid productivity reaching a value of 229.9 mg/L 47 h after inoculating the bioreactor, which represents the stationary phase of the growth. These results agreed with those reported by Malisorn and Suntornsuk [52] for *Rhodotorula glutinis* DM28 while the highest β-carotene production was 201 µg/L after 24 h under the optimized condition of temperature of 30 °C, pH of 6, and dissolved oxygen of 80% in a stirred tank reactor. Meanwhile, Saenge et al. [53] achieved a maximum total carotenoid production of 125.75 mg/L using batch fermentation and 180.20 mg/L after 120 h using fed-batch fermentation in a 2 L stirred tank bioreactor. The increase in carotenoid production is due to the high aeration rate which agreed with the findings of Varmira et al. [32,54].

## 5. Conclusions

Carotenoids, a group of metabolites produced by different organisms, including *Rhodotorula*, have gained massive attention due to their medicinal and industrial benefits for human life. First, carotenoids from *Rhodotorula* ATL72 were extracted and characterized to detect their chemical structure and molecular weight. Then, a sequence of optimization processes was completed using Design of Experiments tools to define the optimum conditions for yeast growth and carotenoid production. The optimum carotenoid production was gained when incubated at 6.7 pH, 27.5 °C, and 3.5% NaCl at 180 rpm supplemented with malt extract, fructose, urea, and yeast extract as the best media components for optimizing the carotenoid production. The scaling up process from shake flask to 1 L bioreactor followed by another scaling up from a 1 L bioreactor to a 7 L bioreactor showed a remarkable increase in the carotenoid production.

## Figures and Tables

**Table 1:** Variables and their levels used for the CCD experiment for temperature, pH, and salinity.

Variables	Levels				
	−2	−1	0	+1	+2
Temperature (°C)	22	25	30	35	38
pH	5.3	6	7	8	8.6
Salinity (%)	0.7	2.5	5	7.5	9.2

**Table 2:** Factors and their levels of the performed CCD for optimization of media components.

Factors (g/L)	Levels				
	−2	−1	0	+1	+2
Malt Extract	2.6	4	6	8	9.3
Fructose	2.6	4	6	8	9.3
Urea	2.6	4	6	8	9.3

**Table 3:** Matrix and responses for the CCD experiment for temperature, pH, and salinity.

Runs	Variables			Responses	
	Temp. °C	pH	Salinity %	Growth (OD_600_)	Carotenoid Conc. (mg/L)
1	35	6	7.5	0.332	4.17
2	30	7	5	0.658	17.23
3	35	8	2.5	0.22	5.82
4	25	6	7.5	0.696	11.34
5	25	6	2.5	0.805	18.8
6	35	6	2.5	0.334	8.95
7	30	7	5	0.658	17.23
8	30	7	5	0.789	22.91
9	30	7	9.2	0.045	1.79
10	30	7	5	0.789	22.91
11	35	8	7.5	0.66	6.34
12	30	8.68	5	0.63	11.26
13	21.5	7	5	0.677	15.97
14	25	8	7.5	0.742	13.35
15	25	8	2.5	0.586	17.38
16	30	7	5	0.866	25.14
17	38	7	5	0.081	4.25
18	30	7	5	0.866	25.14
19	30	7	0.7	0.857	22.16
20	30	5.31	5	0.7	16.64

**Table 4:** Analysis of variance of the experiment of CCD for optimization of temperature, pH, and salinity, the significant values are shown in Bold.

Variables	Reponses					
	OD_600_			Carotenoid Conc.		
	Sum of Squares	*F* Value	*p*-Value	Sum of Squares	*F* Value	*p*-Value
**A: temp**	0.3824	10.42	**0.009**	223.93	16.62	**0.002**
**B: pH**	0.0004	0.01	0.916	6.64	0.48	0.503
**C: salinity**	0.0567	1.55	0.242	183.12	13.59	**0.004**
**A^2^**	0.1898	6.12	**0.033**	197.61	19.37	**0.001**
**B^2^**	0.0028	0.22	0.648	94.02	8.99	**0.013**
**C^2^**	0.1424	3.88	**0.077**	186.64	13.84	**0.004**
**AB**	0.0187	0.51	0.492	0.30	0.02	0.884
**AC**	0.0191	0.52	0.487	6.53	0.48	0.502
**BC**	0.0624	1.70	0.221	9.53	1.70	0.420

**Table 5:** The 20 different media components and their effects on the growth of yeast and carotenoid concentrations through Plackett–Burman design for the isolated yeast.

Run	Variables															Responses
	Glucose	Peptone	Sucrose	Urea	Glycerol	Malt Extract	Fructose	NH_4_NO_3_	Molasses	NH_4_Cl	Whey	Yeast Extract	Potato Ex.	Dummy	Growth (O.D_600_)	Carotenoids Conc. (mg/L)
1	−1	−1	−1	−1	−1	−1	−1	−1	−1	−1	−1	−1	−1	−1	0.001	2.4
2	+1	−1	+1	+1	+1	+1	−1	−1	+1	+1	−1	+1	+1	−1	0.035	3.4
3	+1	+1	+1	−1	−1	+1	+1	−1	+1	+1	−1	−1	−1	−1	0.037	3.4
4	−1	+1	−1	+1	+1	+1	+1	−1	−1	+1	+1	−1	+1	+1	0.072	4.3
5	+1	−1	+1	−1	+1	+1	+1	+1	−1	−1	+1	+1	−1	+1	0.039	2.6
6	−1	−1	+1	−1	+1	−1	+1	+1	+1	+1	−1	−1	+1	+1	0.010	5.1
7	+1	+1	−1	−1	−1	−1	+1	−1	+1	−1	+1	+1	+1	+1	0.003	1.4
8	−1	+1	+1	−1	−1	−1	−1	+1	−1	+1	−1	+1	+1	+1	0.01	2.9
9	−1	+1	−1	+1	−1	+1	+1	+1	+1	−1	−1	+1	+1	−1	0.089	4.9
10	+1	+1	−1	+1	+1	−1	−1	−1	−1	+1	−1	+1	−1	+1	0.001	2.5
11	−1	−1	−1	+1	−1	+1	−1	+1	+1	+1	+1	−1	−1	+1	0.026	3.4
12	+1	+1	+1	+1	−1	−1	+1	+1	−1	+1	+1	−1	−1	−1	0.005	1.7
13	+1	+1	−1	−1	+1	+1	−1	+1	+1	−1	−1	−1	−1	+1	0.018	2.1
14	−1	+1	+1	−1	+1	+1	−1	−1	−1	−1	+1	−1	+1	−1	0.009	2.6
15	+1	−1	−1	−1	−1	+1	−1	+1	−1	+1	+1	+1	+1	−1	0.007	2
16	+1	−1	+1	+1	−1	−1	−1	−1	+1	−1	+1	−1	+1	+1	0.019	3.5
17	−1	−1	−1	−1	+1	−1	+1	−1	+1	+1	+1	+1	−1	−1	0.024	1.5
18	+1	−1	−1	+1	+1	−1	+1	+1	−1	−1	−1	−1	+1	−1	0.007	2.2
19	−1	−1	+1	+1	−1	+1	+1	−1	−1	−1	−1	+1	−1	+1	0.040	2.4
20	−1	+1	+1	+1	+1	−1	−1	+1	+1	−1	+1	+1	−1	−1	0.012	1.4

**Table 6:** Statistical analysis of PBD for optimizing media components for the growth of yeast and carotenoid conc.

Variable	Dry Weight(g/L)				Carotenoid Conc. (mg/L)			
	Effect	Coeffect	*T*-Value	*p*-Value	Effect	Coeffect	*T*-Value	*p*-Value
Glucose	0.01233	0.006165	1.58	0.0158	0.61	0.3	1.61	0.130
Peptone	0.00495	0.002475	0.63	0.546	0.13	0.065	0.28	0.794
Urea	0.01487	0.007435	1.9	0.098	0.37	0.185	0.79	0.468
Malt extract	0.02835	0.014175	3.63	0.008	0.65	0.325	1.72	0.109
Fructose	0.018630	0.009315	2.39	0.048	0.33	0.165	0.7	0.515
Molasses	0.00823	0.004115	1.05	0.327	0.45	0.225	0.96	0.383
Yeast extract	0.00533	0.002665	0.68	0.517	0.57	0.285	1.51	0.155
Whey	0.00307	0.001535	0.39	0.706	0.69	0.345	1.83	0.091
Potato extract	0.00585	0.002925	0.75	0.478	0.89	0.454	2.36	0.035
NH4NO3	0.00175	0.000875	0.22	0.829	0.09	0.045	0.19	0.865
Sucrose	0.00323	0.001615	0.41	0.691	0.23	0.115	0.49	0.646
NH4CL	0.00105	0.000525	0.11	0.914	0.47	0.235	.1.24	0.236
Glycerol	0.00097	0.000485	0.11	0.920	0.03	0.015	0.06	0.952

**Table 7:** Matrix and responses of the CCD for the significant media components.

Run	Variables			Responses	
	Malt Extract g/L	Fructose g/L	Urea g/L	Dry Wt. g/L	Carotenoid Conc. mg/L
1	4	8	8	0.015	23.35
2	6	6	6	0.015	21.19
3	6	6	6	0.015	16.04
4	6	2.6	6	0.014	20.37
5	4	8	4	0.017	12.83
6	6	9.3	6	0.021	16.71
7	6	6	6	0.015	15.07
8	4	4	8	0.013	14.85
9	8	4	8	0.01	11.41
10	8	4	4	0.017	11.86
11	4	4	4	0.017	16.19
12	6	6	6	0.015	17.08
13	6	6	6	0.015	15.22
14	8	8	4	0.019	12.68
15	9.3	6	6	0.024	24.62
16	6	6	9.3	0.012	10.97
17	6	6	2.6	0.012	19.77
18	2.6	6	6	0.017	13.35
19	8	8	8	0.01	11.49
20	6	6	6	0.016	17.67

**Table 8:** Analysis of variance of the CCD experiment for the calculated responses.

Variable	Responses					
	Growth (Dry Weight)			Carotenoids (mg/L)		
	Sum of Squares	*f*-Value	*p*-Value	Sum of Squares	*f*-Value	*p*-Value
A: malt extract	0.000002	0.32	0.583	0.05	0.000	0.965
B: fructose	0.000018	2.41	0.152	0.001	0.000	0.995
C: urea	0.000035	4.69	0.056	3.859	0.16	0.701
A^2^	0.000036	4.07	0.071	0.18	0.000	0.986
B^2^	0.000004	0.31	0.592	0.038	0.02	0.893
C^2^	0.000034	4.54	0.059	24.394	0.99	0.344
AB	0.000000	0.00	1.00	2.247	0.09	0.769
AC	0.000012	0.00	1.00	14.634	0.59	0.459
BC	0.000000	1.65	0.227	15.457	0.63	0.447

**Table 9:** The predicted experiments for the optimal interaction between the media components.

Run	Variables			Responses	
	Malt Ex	Fructose	Urea	OD_600_	Carotenoid Conc. mg/L
1	9.3	2.6	2.6	1.54	13.4
2	2.6	9.3	9.3	1.83	9.7
3	2.7	6	9.3	1.70	11.19
4	8.8	9.3	8.5	1.72	11.9
5	3.7	7.7	9	3.77	24.6

## Data Availability

The data supporting this study are included within the article.
